# Factors Influencing Multi-vehicle Collisions Following Sudden Fatal Health Problems in Drivers

**DOI:** 10.7759/cureus.80438

**Published:** 2025-03-11

**Authors:** Hitomi Kataoka, Masahito Hitosugi, Arisa Takeda, Marin Takaso, Mineko Baba, Mami Nakamura

**Affiliations:** 1 Department of Legal Medicine, Shiga University of Medical Science, Otsu, JPN; 2 Center of Integrated Medical Research, Keio University School of Medicine, Tokyo, JPN

**Keywords:** driving, forensic autopsy, motor vehicle collision, pre-crash technology, sudden death

## Abstract

Introduction: To prevent motor vehicle collisions caused by drivers' health problems, proactive safety measures against impaired driving should be promoted. We assessed the status of both motor vehicles and drivers immediately after sudden and fatal changes in the health of motor vehicle drivers. We also evaluated the factors contributing to multi-vehicle collisions where several people were injured or killed.

Methods: From 70 forensic autopsy cases performed between 1998 and 2023, we found 68 cases in which a vehicle collided with something after the driver experienced a sudden and fatal change in their health. Data about both drivers and vehicles were analyzed.

Results: Heart disease was the most common cause of death (52 drivers), followed by aortic disease (seven) and cerebrovascular disease (five). In total, 25 drivers collided with objects at ≥40 km/hour, and 35 collided at <40 km/hour; 17 drivers collided with other vehicles, and 51 with something other than a vehicle. Multivariable logistic regression analysis suggested that maneuvering forward and to the left (toward the side of the road) rather than straight ahead was a significant predictive factor for avoiding collision with other vehicles (odds ratio of 0.026).

Conclusion: The findings from our study aim to enhance the development of driver monitoring systems and pre-crash safety technologies, with the ultimate goal of reducing the number of casualties in motor vehicle collisions.

## Introduction

An acute health change while driving is one of the major causes of vehicle collisions. Approximately 1.19 million people die and between 20 and 50 million people are injured in motor vehicle collisions (MVCs) annually worldwide [1]. Road traffic injuries cause huge economic losses to individuals, their families, and nations as a whole; the cost is approximately 3% of the gross domestic product in most countries [1]. The World Health Organization has therefore recommended that all governments act to address road safety in a holistic way [1]. In Japan, the government has set an objective of a safe traffic society, where no collisions occur, and has issued a Basic Plan for Traffic Safety that is revised every five years. The 11th Traffic Safety Basic Plan, which started in 2021 and runs until 2025, comprises concrete behavioral goals: fatalities of 2,000 or less and serious injuries of 22,000 or less. Analyzing the trends and characteristics of MVCs should enable effective preventable measures to be developed [2].

In 2023, there were 2,678 fatalities from MVCs in Japan, 2.6% more than in the previous year, and 27,636 people were severely injured, 6.2% more than in the previous year [3]. Pedestrians were the biggest group killed or severely injured, followed by motor vehicle passengers [3]. In Japan, population aging and low birth rates are serious nationwide problems. Consequently, the number of older drivers has also increased. In 2022, 23.8% of driving license holders were 65 years old or over [4]. Because many older people have health problems, such as hypertension, diabetes, glaucoma, and lower back pain, sudden changes in their health can therefore be a cause of MVCs.

An acute health change while driving is one of the major causes of vehicle collisions. According to a report from Finland, incapacity of the driver caused by sudden illness was the immediate cause of collisions in 10.3% of all fatal MVCs [5,6]. A previous study carried out a retrospective analysis among commercial drivers who had suffered from sudden illness while driving [7]. It found that cerebrovascular disease was the most common cause of sudden illness (28.4%), followed by cardiac and aortic disease (26.1%), syncope (8.5%), and digestive disease (8.1%) [7]. Cerebrovascular and cardiac disease often occur while driving. Inamasu et al. examined the onset of stroke in people attending the emergency department at a hospital between 2011 and 2016 [8]. They reported that 4.0% of strokes occurred while driving a motor vehicle [8]. Inoue et al. examined post-stroke patients undergoing rehabilitation and found that 2.8% had their stroke while driving [9]. A prospective study among patients admitted to an emergency department found that the frequency of acute coronary syndrome while driving was 4.0% [10]. In addition to these common diseases, transient physiological changes, such as vasovagal syncope, also occur while driving and sometimes have serious consequences [11]. A substantial number of these drivers had a collision immediately after experiencing these health problems. In a study among professional drivers suffering from sudden illness while driving, 64.7% lost control of their vehicles and caused MVCs [7]. After a stroke, 49.4% of drivers could not continue driving, and 12.9% had MVCs [8]. Other reports on people having a stroke while driving suggested that 16.7% suffered from subsequent MVCs with symptoms of consciousness disorder or muscle weakness [8].

Sudden death while driving has been reported from forensic autopsies. The major cause of sudden death while driving was ischemic heart disease [12]. Among drivers suffering from fatal cardiac diseases, aortic disease, or cerebrovascular diseases, 73.5% could not take evasive action such as steering or braking [13]. Preventing MVCs due to health problems in drivers requires active safety measures to prevent impaired driving.

Recently, autonomous driving techniques have been developed, and collision avoidance systems have been equipped for new vehicles. These systems are based on monitoring the area around the vehicle through a combination of cameras and sensors to detect potential threats or danger and then activate a warning. However, when the drivers suffer from acute health changes and cannot take corrective actions, the system has to take necessary actions to prevent subsequent collisions. Therefore, for the development of autonomous driving, the situation in which the driver suffers from sudden health problems while driving has to be assumed. Furthermore, if the collision is not avoided due to the driver's acute health changes, the system has to decide how to minimize the number of people involved.

The present study aims to provide valuable information to develop a collision avoidance system due to drivers' health problems. To establish what technologies are needed, it is important to understand the kinematics of drivers immediately after the onset of sudden health changes and the running state of the vehicle in these cases. Therefore, the objectives of this study were to examine the status of both motor vehicles and drivers immediately after the drivers had experienced sudden health changes and to identify the factors that contribute to vehicle collisions involving several vehicles, which may lead to greater numbers of injuries or fatalities.

## Materials and methods

Samples

**Figure 1:**
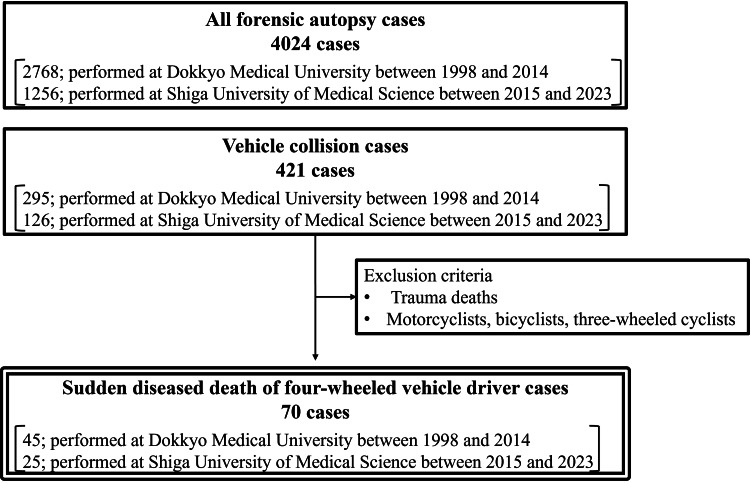
Case selection and exclusion criteria.

We examined the forensic autopsy cases performed at Dokkyo Medical University between 1998 and 2014 and Shiga University of Medical Science between 2015 and 2023 to identify those involving the sudden death of drivers of four-wheeled vehicles. Forensic autopsy was performed for cases in which the cause of death was not determined with the investigations at the emergency room or the mechanism of injuries was not fully confirmed. Among all forensic autopsy cases, first, we selected cases in which the victim was involved in a vehicle collision. Subsequently, after excluding trauma deaths and cases in which the victims were motorcyclists, bicyclists, and three-wheeled cyclists, sudden disease deaths while driving a vehicle were selected for analysis (Figure 1).

No comprehensive statistics exist regarding sudden disease death while driving a vehicle. Although case reports have frequently been presented, the prevalence of these cases has not been accurately established. Therefore, it is difficult to calculate the sample size for our study.

Information

The autopsy records and information files provided by the police in each case were retrospectively reviewed. The following information was collected: (1) the general characteristics of the drivers: age, sex, height, weight, and body mass index (BMI). (2) The characteristics of the situation at the time of the driver's acute health change (incident): time and place of the incident and the type of vehicle. The time of the incident was then classified as day (between 6 a.m. and 6 p.m.) and night (between 6 p.m. and 6 a.m.). For the place of the incident, we examined the road characteristics and categorized it as a road with a center strip and a narrow road without a center strip. The type of vehicle was classified as a normal passenger vehicle, a mini-vehicle (Kei-car), a mid-sized truck weighing 5-11 tons, or a large truck weighing 11 tons or more. (3) Detailed information about the subsequent collision: the movement of the vehicle before and immediately after the collision. We examined the trajectory of the vehicle immediately before the collision. Subsequently, we classified the direction of the travel as straight ahead, forward-right, or forward-left. In Japan, owing to the left-hand traffic, moving forward-right means toward oncoming traffic. For each collision, the collided object was examined and classified as a vehicle or other objects, such as a pole, curb, building, or tree. The collision velocity was determined by the police investigation and categorized as ≥40 km/hour or less. (4) State of the driver when found: the time between the onset of the incident and being found; the presence of other passengers in the vehicles; the driver's consciousness level (Glasgow Coma Scale (GCS)); the driver's use of a seatbelt; and the posture of the driver when found. The authors determined the GCS of the patients based on the driver's condition at the scene (state of eye-opening and verbal and motor responses to paramedic personnel). The posture of the driver was categorized as upright position, leaning forward, face up, leaning right, leaning left, and others. (5) Cause of death: final diagnosis obtained from forensic autopsy.

Statistical analysis

The data were summarized as values with proportions or frequencies for categorical variables. We used mean ± standard deviation (SD) for values that followed a normal distribution to summarize continuous variables. We divided the cases whether the vehicle collided with other vehicles or not. Subsequently, the prevalence regarding the time of collision, type of road, type of vehicle, direction of travel, collision velocity, and consciousness level of the driver was compared between these two groups. Chi-square tests and Fisher's exact tests were used for the analyses. We used multivariable logistic regression analysis to identify which variables were independently associated with multi-vehicle collisions. The analyses used IBM SPSS Statistics for Windows, Version 23 (Released 2015; IBM Corp., Armonk, New York). A p-value < 0.05 was considered statistically significant.

Ethical consideration

This study was conducted using anonymized existing data without intervention, such as the information from the autopsy records. The consent of the kin was obtained via the opt-out method based on the Personal Information Protection Act, Japan. Opportunities to opt out were provided through the publication of study details on the Shiga University of Medical Science website. This study was approved by the Ethics Committee of Shiga University of Medical Science (approval no.: R2014-010).

## Results

General aspects

We collected data on 70 drivers (65 men and five women) for analysis. Of these, two drivers had stopped their vehicles immediately after experiencing the health problem. The remaining 68 drivers were included in the analysis. Their ages ranged from 27 to 88 years (mean ± SD of 59.0 ± 14.4 years). The mean height was 167.0 ± 7.8 cm, the mean weight was 71.4 ± 17.2 kg, and the mean BMI was 25.3 ± 4.8 kg/m^2^ (Table 1). No one received psychiatric management immediately before the incident.

**Table 1:** Characteristics of the drivers included in the study BMI: body mass index; SD: standard deviation

Characteristics of drivers	
Age (mean ± SD)	59.0±14.4
Sex (male/female)	63/5
BMI (mean ± SD)	25.3±4.8
Duration between the incident and being found (n)	
≤ 10 min	14 (21%)
10–30 min	34 (50%)
> 30 min	11 (16%)
Unknown	9 (13%)
Seatbelt use (n)	
Used	41 (60%)
Not used	17 (25%)
Unknown	10 (15%)
Posture of the driver (n)	
Upright position	17 (25%)
Leaning forward	3 (4%)
Face up	10 (15%)
Leaning right	2 (3%)
Leaning left	21 (31%)
Others	15 (22%)
Cause of death (n)	
Heart disease	52 (76%)
Aortic disease	7 (10%)
Cerebrovascular disease	5 (7%)
Others	4 (6%)

Characteristics of the incident

Acute health changes (incidents) while driving occurred during the day (between 6 a.m. and 6 p.m.) for 47 drivers, at night (between 6 p.m. and 6 a.m.) for 16 drivers, and at an unknown time for five drivers. The incidents occurred when driving on roads with a center strip for 47 drivers and on a narrow community road without a center strip for 16 drivers. One incident occurred in a parking area, and the details were unknown for four drivers. Overall, 45 drivers were driving passenger vehicles, including 42 normal passenger vehicles and three mini-vehicles (Kei-car). The remaining 23 drivers were driving trucks, including 19 mid-sized ones weighing 5-11 tons, three large ones weighing 11 tons or more, and one with an unknown size.

Movement of the vehicle

Immediately after the driver's health issue, all 68 vehicles continued to run. Of these, 22 vehicles moved forward and to the left (toward the roadside), seven went straight ahead, 36 moved forward and to the right (toward any oncoming traffic), and three moved in an unknown direction. All 68 vehicles collided with something: 51 with objects other than vehicles, such as poles, curbs, buildings, and trees, and 17 with other vehicles (Table 2). No vehicles collided with pedestrians. In total, 25 collisions happened at ≥40 km/hour and 35 at <40 km/hour (Table 2). The collision velocity could not be properly assessed in eight cases.

**Table 2:** Comparison of aspects of the collision Differences with p < 0.05 were considered statistically significant.

Variable	Vehicles (n=17)	Others (n=51)	p-value
Time of collision			
Day	13 (76%)	34 (74%)	0.56
Night	4 (24%)	12 (26%)	
Type of road			
Road with a center strip	14 (93%)	33 (69%)	0.051
Narrow community road	1 (7.0%)	15 (31%)	
Type of vehicle			
Passenger vehicle	12 (71%)	33 (65%)	0.66
Truck	5 (29%)	18 (35%)	
Direction of travel			
Straight ahead	1 (6.0%)	6 (13%)	0.33
Forward-right	12 (71%)	24 (50%)	
Forward-left	4 (24%)	18 (38%)	
Collision velocity			
≥ 40 km/hour	6 (60%)	19 (38%)	0.17
< 40 km/hour	4 (40%)	31 (62%)	
Consciousness level of the driver			
GCS = 3	14 (82%)	46 (90%)	0.38
GCS > 3	3 (18%)	5 (10%)	

Status of the drivers when found

We also examined the drivers' status when found. A total of 14 drivers were found within 10 minutes of the incident, 48 within 30 minutes, and 11 after more than 30 minutes (Table 1). In three cases, passengers were found in their vehicles. Most drivers (88.2%) had a GCS of three; there was also one driver with a score of four, two of 12, two of 13, and three of 15. Overall, 41 drivers were wearing seatbelts, 17 were not, and in 10, the situation was unknown (Table 1). The posture of the drivers is also summarized in Table 1. The most common postures were leaning left and upright.

Cause of death

A forensic autopsy established the cause of death for each driver. The most common was heart disease, affecting 52 drivers (ischemic heart disease in 44, acute heart failure in six, and chronic heart failure and dilated cardiomyopathy, each in one person). This was followed by aortic disease in seven drivers (rupture of aneurysm in four and dissection in three); cerebrovascular disease in five drivers, all cerebral hemorrhage; and other problems in four, including pulmonary thromboembolism, liver cirrhosis, lung cancer, and cholangitis.

Comparison of multi-vehicle collisions and other collisions

Generally, when collisions involving multiple vehicles occur, more people are injured or killed. To identify the characteristics of incidents involving multiple vehicles, we compared cases where vehicles had collided with other vehicles and those involving no other vehicles, as shown in Table 2. There were no significant differences in six items between the two groups.

Variables associated with collisions with other vehicles

We used multivariable logistic regression analysis to identify variables that were independently associated with collisions with other vehicles. We considered that the time of the collision, collision velocity, and direction of vehicle movement immediately after the incident were the important items for collisions involving other vehicles. Because these items can be easily monitored in the vehicle and may facilitate the implementation of a novel system to avoid collisions due to drivers' health problems, we selected these three items as predictive variables. Moving forward and to the left (i.e., toward the roadside) compared with straight ahead was a significant factor in avoiding a collision with other vehicles (odds ratio of 0.026) (Table 3). This analysis was not affected by multicollinearity with variance inflation factors of <1.29. The Hosmer-Lemeshow test indicated a good fit (p=0.638), and Nagelkerke's R^2^ was 0.345.

**Table 3:** Results of multivariable logistic regression analysis Differences with p < 0.05 were considered statistically significant. Ref: reference

Variable	Odds ratio	95% Confidence interval	p-value
Time of the collision (ref: day)	2.388	0.526-10.843	0.260
Collision velocity (ref.: ≥40 km/hour)	0.891	0.208-3.814	0.876
Direction of travel (ref.: straight ahead)			
Forward-right	0.192	0.029-1.293	0.090
Forward-left	0.026	0.002-0.371	0.007

## Discussion

This study clarified the status of both motor vehicles and drivers immediately after a sudden change in the driver's health. In 37% of the cases, collision velocity exceeded 40 km/hour. A previous study on people experiencing cardiac arrest while driving found that most vehicles were moving at a slow speed before the incident [14]. It also suggested that the affected drivers might have released the accelerator pedal after cardiac arrest. However, that study was not based on police investigations, and collision velocity was not correctly determined. Our study confirmed that high-speed collisions following a sudden change in the driver's health could occur in many cases. We, therefore, confirmed that serious MVCs with multiple casualties can occur even in collisions due to drivers' health problems. We also confirmed conditions that were associated with multi-vehicle collisions. We found that moving forward and to the left was a significant predictor in avoiding multi-vehicle collisions because traffic drives on the left-hand side in Japan. If pedestrians are traversing the area, the vehicle may collide with them. However, we found no cases of this type of collision, suggesting that the possibility of pedestrian-vehicle contact after a sudden change in the driver's health is relatively low. The use of safety barriers would also help to keep pedestrians safe. To prevent serious MVCs, it is desirable to develop pre-crash safety technology that moves the vehicle away from oncoming traffic and stops it immediately after a sudden change in the driver's health.

Most drivers were leaning right, left, or forward (38%) when found. In these positions, safe driving maneuvers are difficult. In total, 64 drivers had GCS scores of three or four when found. If it were possible to monitor drivers' positions or levels of consciousness, subsequent collisions could be avoided. Therefore, improvements in driver monitoring systems are required. Our results may contribute to the development of these technologies.

This technology may have some possibilities for saving the lives of drivers affected by these health problems. We found that 21% of drivers were found within 10 minutes of the incidents. For people experiencing cardiac arrest while driving, seven of 17 patients exhibited asystole, and 10 showed ventricular fibrillation on admission. However, eight patients had a favorable outcome six months after the incidents [14]. Therefore, finding the affected drivers as soon as possible after the incidents is a priority. An automatic notification system for MVCs has recently been developed. In the European Union, the implementation of this system in new models of cars was mandated in 2018. This is still under consideration elsewhere. The system may contribute to the rescue of drivers in accidents, especially those who have experienced sudden health problems. However, this system does not operate if a driver voluntarily stops the vehicle or if the vehicle stops without a collision. In these cases, a driver monitoring system would be needed to detect health abnormalities and call for emergency help.

Most of the drivers in this study were men, with a mean age of 59.0 years. These values were consistent with previous reports on autopsied sudden deaths while driving [5,6,12,13]. We found that 76.5% of drivers had died of heart disease. An autopsy study in Finland involving drivers suffering from sudden health problems found that the most common cause of death was cardiovascular disease (70.0%) [5]. Another autopsy study from Canada found that cardiovascular disease was the leading cause of death in drivers who died in MVCs [6]. The study also suggested that coronary artery diseases were associated with a 9% probability of fatal MVCs. A report on autopsies among people who died suddenly while driving a vehicle in Japan found that ischemic heart disease was the leading cause of death, accounting for 64.7% of the total [13]. Heart disease was also the most common condition among patients admitted to an emergency department in Japan because of cardiac arrest while driving, accounting for 82.4% of cases [14]. Hitosugi et al. reviewed professional drivers who had been ordered to stop driving due to health problems [7]. Among those who died while driving, cardiac and aortic diseases were the most frequent problems (57.9%). Several studies have, therefore, shown that the leading cause of death among people who experienced sudden death while driving a motor vehicle was heart disease, especially ischemic heart disease. However, the rate of heart disease among sudden deaths while driving depends on the characteristics of the cases.

Stroke or rupture of an aortic aneurysm while driving is also often reported [9,11,13,14]. For these cases, whole-body computed tomography can provide a diagnosis in the emergency room. Autopsies may not be performed if the collision circumstances are confirmed or no collisions occur. However, the diagnosis of sudden cardiac deaths is difficult because of a lack of specific findings before an autopsy. Studies based on autopsies are, therefore, more likely to show heart disease as the dominant cause of death. In the future, to clarify the frequency of fatal diseases while driving a motor vehicle, population-based studies using autopsies are required.

Various diseases can cause sudden incapacity while driving [7-9,11]. If these diseases themselves do not become fatal, they may lead to MVCs, and these may cause severe injuries. Forensic pathologists must, therefore, consider that various diseases may cause MVCs, even in cases of trauma death. In the future, better driver monitoring systems may support the diagnosis of forensic pathologists that the inability to drive was due to acute health changes.

This study had some strengths. First, as we collected the 68 real-world collision cases due to drivers' health problems, some novel findings were clarified: collision velocity exceeded 40 km/hour in 37% of cases; the posture of the driver was wide-ranging, and it is difficult to judge the driving difficulty from the post-collision posture. Second, as the present study aimed to provide valuable information to develop a collision avoidance system due to drivers' health problems, we could elucidate that moving toward the side of the road rather than straight ahead was a significant predictive factor for avoiding collisions with other vehicles. This result might contribute to developing an autonomous driving system that acts in situations where the driver suffers from sudden health problems while driving.

This study had some limitations. First, because the investigation of the MVCs was performed by the police, there may be selection bias and observer bias. The cases included in the present study had been autopsied for suspecting acute health changes while driving. However, owing to the observer bias, there may be some cases in which a forensic autopsy was not performed because the cause of the collision had been determined as the human error of the driver. Therefore, the present samples were not representative samples of a particular area due to selection bias. Second, the people concerned died in suburban areas in Japan. In these areas, forensic autopsy is generally performed for MVCs in which drivers' health problems were suspected as a cause. However, we did not find any cases of severe collisions involving many seriously or fatally injured people. If data were collected from urban areas with high population density, more data on serious MVCs may be obtained. Future studies should, therefore, use a similar approach in larger cities. Third, our study was based on forensic autopsies, and therefore, most of the drivers included experienced sudden cardiac deaths. The kinematics of the vehicle depend on the characteristics of the driver at the time of the incident. Therefore, this kind of research should also be carried out among drivers suffering from other diseases. As mentioned before, population-based studies are necessary to confirm effective pre-crash technologies. Fourth, the sample size was relatively small. We collected data on autopsies of drivers experiencing sudden onset of health changes while driving from autopsy records over 25 years in two facilities. These autopsy cases were rare, and finding enough data from one facility was difficult. In the future, a multicenter registration system is required. Fifth, the psychological status of the driver immediately before the collision could not be evaluated. In this study, although no one had received psychiatric management immediately before the incidents, detailed information about the psychological status of the driver was not obtained. Because evaluating the drivers' psychological status after death is difficult, this issue would be examined with nonfatal collision cases.

## Conclusions

Our study examined vehicle collisions following the sudden onset of a fatal health problem while driving. Heart disease was the most common cause of death, followed by aortic and cerebrovascular diseases. Multivariable logistic regression analysis suggested that moving forward and to the left (toward the side of the road) rather than straight ahead was a significant predictive factor for avoiding collisions with other vehicles. The authors believe that the results of this study may contribute to the reduction of casualties from MVCs caused by drivers' health problems.
